# *Arthrobotrys thaumasia* and *A. musiformis* (Fungi: Orbiliales) as Natural Potential Enemies of *Haemonchus contortus* (Nematoda: Trichostrongylidae)

**DOI:** 10.3390/pathogens15080784

**Published:** 2026-07-23

**Authors:** Mariana Cervantes-Martínez, Rosa Isabel Higuera-Piedrahita, Héctor Alejandro de la Crúz-Crúz, Gustavo Pérez-Anzúrez, María Eugenia López-Arellano, Agustín Olmedo-Juárez, Edgar Jesús Delgado-Núñez, Ana Yuridia Ocampo-Gutiérrez, Pedro Mendoza-de Gives

**Affiliations:** 1 Laboratory of Helminthology, National Centre of Disciplinary Research in Animal Health and Safety, Instituto Nacional de Investigaciones Forestales, Agrícolas y Pecuarias (INIFAP), Boulevard Paseo Cuauhnahuac No. 8534, Col. Progreso, Jiutepec 62556, Morelos, Mexico; cmmo211087@upemor.edu.mx (M.C.-M.); tavopzaz@gmail.com (G.P.-A.); mlopez_arellano@hotmail.com (M.E.L.-A.); aolmedoj@gmail.com (A.O.-J.); 2 Faculty of High Studies-Cuautitlán, National Autonomous University of Mexico, Carretera Cuautitlán-Teoloyucan km 2.5 San Sebastián Xhala, Cuautitlán Izcalli 54714, Estado de México, Mexico; rhiguera05@comunidad.unam.mx (R.I.H.-P.); delacruz@unam.mx (H.A.d.l.C.-C.); 3 Faculty of Agricultural, Livestock and Environmental Sciences, Autonomous University of the State of Guerrero, Iguala de la Independencia 40040, Guerrero, Mexico; edgarjezus@gmail.com

**Keywords:** nematode-trapping fungi, *Arthrobotrys*, biocontrol, nematode/fungi interaction, nematocidal activity

## Abstract

*Haemonchus contortus* (*Hc*) is a parasitic nematode affecting sheep. *Arthrobotrys* is a group of nematophagous fungi (NF) that capture nematodes with specialized traps. This study evaluated the in vitro predatory ability of *Arthrobotrys thaumasia* (*At*) and *A. musiformis* (*Am*), as well as the nematocidal activity of their liquid culture filtrates against *Hc*. NF isolates were obtained by sprinkling soil onto agar plates containing nematodes. Isolates were identified morphologically and molecularly. The predatory activity (PA) was tested on agar plates inoculated with nematodes and fungi; plates inoculated only with nematodes served as controls. After 10 days at 18 °C and 28 °C, larvae were counted. For nematocidal activity (NA) of LCFs, fungi were grown in Czapek–Dox (CzDB) and potato dextrose broths. Two concentrations (100 mg/mL and 130 mg/mL) were tested. Nematodes and LCFs were incubated for 48 or 72 h. PA was analyzed using unpaired Student’s *t*-tests, and LCFs were evaluated by ANOVA with Tukey’s post hoc test. Taxonomic procedures confirmed the isolates as *Am* and *At*. For PA, effectiveness was 74.64% for *Am* and 83.53% for *At*. The highest NA of LCF of *Am* in CzDB was 51% at 100 mg/mL and 76.7% at 130 mg/mL. That of the LCF of *At* was 22% at the highest concentration. Both isolates show strong potential as biocontrol agents against haemonchosis.

## 1. Introduction

Gastrointestinal parasitic nematodes (GPNs) pose a major challenge to the livestock industry worldwide [1]. GPN infections lead to a decline in animal health, marked by clinical signs such as anemia, hypoproteinemia, anorexia, weakness, and weight loss, and, in severe cases, this can result in the death of animals, especially young ones whose immune systems are not yet fully developed [2]. Enormous economic losses threaten the livestock industry worldwide [3]. An estimation of approximately €1.8 billion of economic losses in ruminants was made in Europe [4]. Beyond these significant concerns, the primary strategy for controlling GPNs—frequent and continuous administration of chemical anthelmintic drugs—has notable drawbacks. These include the rapid development of anthelmintic resistance among parasites [5,6], the risk of contamination of milk, meat, and animal by-products destined for human consumption [7], and the potential for environmental harm. Drug residues eliminated in the urine and feces of treated animals can contaminate soil, posing risks to beneficial non-target microorganisms [8,9,10]. These disadvantages have given anthelmintic chemotherapy a poor reputation as a control strategy. Nevertheless, chemotherapy with anthelmintic drugs remains the most commonly used control strategy so far. Therefore, exploring new, sustainable alternative methods for controlling GPNs is crucial to avoid the undesirable direct and indirect negative effects associated with anthelmintic chemotherapy [11]. Several alternative control strategies have been investigated, including the use of plants and plant metabolites with nematocidal activity [12], grazing management [13], vaccines [14,15], copper particles [16], and natural nematode antagonists such as nematophagous fungi [17]. Nematophagous fungi are saprobic microorganisms that inhabit soil and exhibit facultative parasitic or predatory behaviour toward nematodes [18]. The genus *Arthrobotrys* is a group of nematode-trapping fungi widely recognized as a natural enemy of both phytoparasitic and animal-parasitic nematodes [18]. The species *Arthrobotrys thaumasia* and *A. musiformis* were selected for this study because *A. thaumasia* is relatively understudied, with only a few reports, yet it appears to have significant potential to control parasitic nematodes important to the livestock industry. In contrast, certain isolates of *A. musiformis* have already demonstrated significant potential as agents against haemonchosis. However, continued exploration of new isolates from this species could reveal even more effective candidates for nematode control, whether through their predatory behaviour or the use of their liquid culture filtrates. These fungi are considered promising biotechnological tools for managing pests of agricultural and livestock importance. The objectives of this research were: (1) to evaluate the in vitro predatory activity (PA) of nematophagous fungi against infective larvae of the sheep parasitic nematode *H. contortus*, and (2) to assess the nematicidal effects of these fungi through bioassays using liquid culture filtrates (LCFs) from two *Arthrobotrys* species.

## 2. Materials and Methods

### 2.1. Location

This study was carried out at the Helminthology Laboratory of the National Centre for Disciplinary Research in Animal Health and Innocuity of the National Institute of Research in Forestry, Agriculture and Livestock (CENID-SAI, SAGAR). The laboratory is located at Carretera Cuernavaca-Cuautla 8534, Progreso, 62574 Jiutepec, Morelos, Mexico.

### 2.2. Biological Material

#### 2.2.1. Nematodes *Panagrellus redivivus*

The free-living nematode *Panagrellus redivivus* was obtained from a pet shop as fish food in Jiutepec, Morelos. This nematode was cultured on an oat medium prepared as follows: 30 g of oatmeal was boiled in 100 mL of distilled water for 5 min, the medium was allowed to cool, and 35 mL of the nematode *P. redivivus* previously filtered in a Baermann funnel was added. The culture was propagated for 7 days at 18–25 °C, during which time nematode larvae and adults were obtained [19]; to separate them from the oats, larvae were filtered through a 74 µm sieve and recovered using the Baermann funnel technique. Finally, they were washed three times with distilled water [20].

#### 2.2.2. Obtaining *Haemonchus contortus* Infective Larvae (L3)

*Haemonchus contortus* nematode larvae were obtained from the infection of a donor hair sheep (Pelibuey). This animal was orally infected with 350 infective larvae (L3)/Kg BW. The *H. contortus* (Hueytamalco strain) obtained from a naturally infected sheep in a sheep farm in Hueytamalco, Puebla, Mexico, was used. It is important to highlight that the management of the parasite egg-donor lamb was carried out in accordance with strict animal welfare standards, preventing unnecessary suffering and adhering to best practices established by INIFAP. Compliance with the Norma Oficial Mexicana NOM-052-ZOO-1995 (http://legismex.mty.itesm.mx/normas/zoo/zoo052-Cancel2016_05.pdf#1#1, accessed on 9 August 2024) and the Ley Federal de Sanidad Animal DOF 07-06-2012 (http://www.diputados.gob.mx/LeyesBiblio/ref/lfsa.htm, (accessed on 9 September 2024)) was rigorously maintained. All procedures in this study followed INIFAP’s ethical guidelines. The confirmation of the infection was performed by counting the number of fecal eggs using the McMaster technique after a pre-patent period of 21 days [20]. After this period, feces infected with *H. contortus* eggs were collected for 24 h. To obtain L3 larvae, a coproculture was mixed with polyurethane foam particles. This was removed daily to allow aeration and good development of the L3 larvae; the culture was incubated for 7 days. They were recovered using the Baermann funnel method [21,22]. These were kept refrigerated at 4 °C until use. Recovered larvae were washed by differential centrifugation for 5 min at 2500× *g*, using a 40% sucrose (commercial sugar) gradient [23]. After centrifugation, the floating ring at the interphase, corresponding to the larval package, was recovered. The material containing the larvae was placed in a 15 mL test tube with sterile distilled water and centrifuged three times to remove any remaining sucrose.

### 2.3. Isolation of Nematophagous Fungi

**Figure 1:**
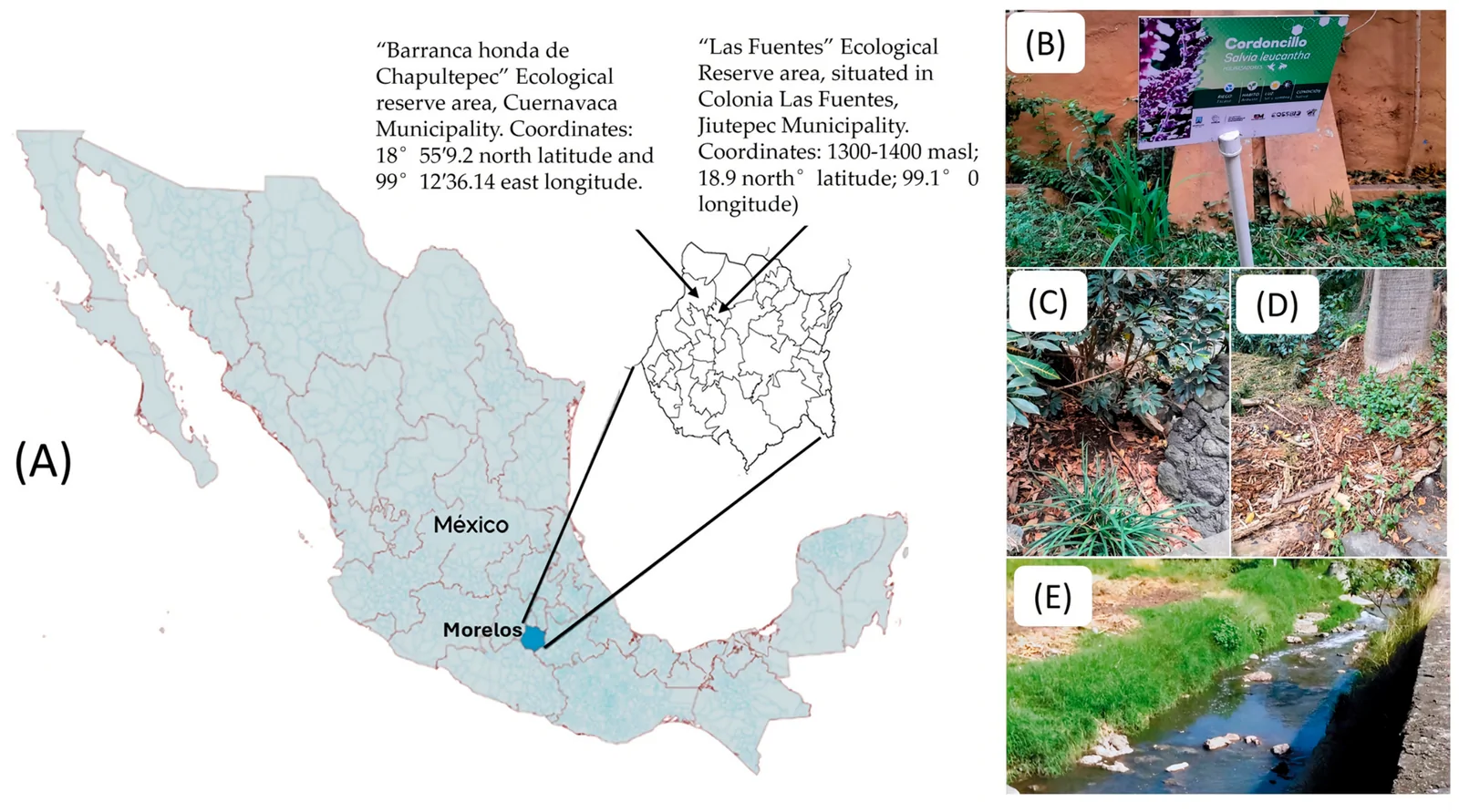
(**A**) displays a map of the Mexican Republic highlighting the Cuernavaca and Jiutepec Municipalities in the State of Morelos. (**B**) shows the sample collection site at Barranca de Chapultepec Natural Reserve, where soil was taken from the base of the “Cordoncillo” plant (*Salvia leucantha*). (**C**,**D**) depict the surrounding vegetation. (**E**) presents the isolation site (beside the river) at the recreational resort center, “Las Fuentes.”

Soil samples were collected from two ecological areas of Mexico. One was isolated from the Natural Protected Area “Parque Estatal Urbano Barranca de Chapultepec”, located in Colonia Chapultepec, Cuernavaca, State of Morelos, Mexico (18°55′9.2 north latitude and −99°12′36.14 east longitude). The other isolate was obtained from “Las Fuentes” Ecological Reserve Area, situated in Colonia Las Fuentes, Jiutepec Municipality, Morelos State, Mexico (Figure 1).

To obtain fungal isolates, 10 g of soil and 50 g of leaf litter were collected from various locations within the study area. Each sample was plated in triplicate using 90 × 15 mm Petri dishes containing 20 g/L of 2% bacteriological agar (Becton Dickinson, Cuautitlán, Mexico). Soil samples were inoculated using the soil sprinkling technique, which involves distributing a small amount (0.3–0.5 g) of soil onto the agar surface [24]. Plates were incubated for 8 days at 18–25 °C. To promote the development of nematophagous fungal structures, an aqueous suspension with approximately 1000 free-living *P. redivivus* nematodes was added to each dish. Fungi were isolated by examining the plates under a light microscope to identify aerial structures characteristic of nematophagous fungi.

### 2.4. Morphological Identification of the Fungi

For morphological identification, a 1 × 1 cm^2^ rectangle of PDA (Bioxon, Cuautitlán, Mexico) was placed on a microscope slide and inoculated with the obtained fungal strain to prepare a microculture. The sample was then transferred to a Petri dish with a wet paper towel at the bottom to maintain its humidity. The slide was incubated at room temperature for 5 days. After incubation, the sample was stained with cotton blue to visualize the fungal structures. Observations were performed under a brightfield microscope (Leica Microsystems, Wetzlar, Germany) at 40× magnification. Under these conditions, trapping devices and predated nematodes were observed, and structures such as conidia, conidiophores and chlamydospores were measured. Morphometric analysis was conducted by measuring fungal structures in 30 microphotographs and comparing them with taxonomic identification tables reported in the literature [25,26].

### 2.5. Molecular Identification of Fungal Isolates

An isolate obtained from Barranca de Chapultepec (designated in our collection as the BCh isolate) and another from the “Las Fuentes” Ecological Reserve Area (designated as the LF Isolate) were cultured in Czapek–Dox broth (Becton Dickinson, Cuautitlán, Mexico) for 7 days at 18–25 °C. After incubation, 10–20 mg of mycelium was harvested and placed in 1.5 mL Eppendorf tubes. DNA extraction was performed using the Wizard^®^ Genomic DNA Purification Kit (Promega, Madison, WI, USA), and the extracted DNA was quantified using an NanoPhotometer NP80 (IMPLEN, Munich, Germany). The DNA served as a template for endpoint PCR using a C1000 Touch^®^ Thermal Cycler (Bio-Rad, Hercules, CA, USA), as described by [27]. Amplification was achieved using ITS4 (5′-GGAAGTAAAAGTCGTAACAAGG-3′) and ITS5 (5′-TCTCCTCCGCTTATTGATATATGC-3′) primers [28]. PCR conditions were: initial denaturation at 94 °C for 3 min; 35 cycles of denaturation at 94 °C for 60 s, annealing at 42 °C for 90 s, extension at 72 °C for 90 s; final extension at 72 °C for 90 s; and cooling to 4 °C. PCR products were separated by 1.5% agarose gel electrophoresis for 70 min at 70 V and visualized with an EC3 Imagen System photo-documenter (UVP Inc. Jena, Germany). Amplicons were purified using a QIAquick gel extraction kit (QIAGEN, Hilden, Germany) and sent to the Institute of Biotechnology at the UNAM (Mexico) for sequencing. Sequence analysis and alignment were performed using the NCBI BLAST database (https://blast.ncbi.nlm.nih.gov/Blast.cgi, accessed on 3 September 2024) [29].

### 2.6. Phylogenetic Analysis

Phylogenetic analysis was conducted to confirm taxonomic identification. A total of 52 sequences from the NCBI database were employed, using *Vermispora fusarina (YXJ13-5)* as an outgroup. Multiple sequence alignment was performed using the CLUSTAL algorithm in MEGA (v11.0.13). The best substitution model (TIM2 + F + G4) was determined with JModelTest software (v2.1.10) and used to construct a phylogenetic tree via the Maximum Likelihood method in IQTree (v1.6.12). Bootstrap support values were obtained using the ultrafast bootstrap method with 10,000 replicates. The resulting tree was visualised in FigTree v1.4.4 and refined with Nitro Pro (v13.9.1).

### 2.7. Predatory Activity Assessment

To evaluate the predatory activity, three treatments were arranged in 60 × 15 mm^2^ Petri dishes containing bacteriological agar (*n* = 10). The treatments consisted of BCh and LF isolates, and a fungus-free control. One agar plug (1 cm diameter) from each of the BCh and LF isolates at 15 days old (in water agar, at 18–25 °C) was transferred to fresh sterile water agar plates for groups 1 and 2, respectively, while group 3 received no fungal inoculation. All plates were incubated at 18–25 °C for 7 days. After this period, 100 μL of an aqueous suspension containing 500 *H. contortus* infective larvae was added to each dish in groups 1 and 2, followed by a further 10-day incubation at 18–25 °C. Larvae from each treatment were then recovered using the Baermann funnel technique [30].

The control group served as the reference, representing 100% larval recovery in the absence of fungal exposure. Larval counts were determined by examining 10 μL aliquots under a light microscope at 5× and 10× magnifications. The percentage reduction in larvae was calculated using Abbott’s formula:

Predatory activity % = [(LRC − LRI)/LRI] × 100

where:

• LRC = larvae recovered from the control group.
• LRI = larvae recovered from the fungus/larvae interaction group.
• The data were analyzed using paired Student’s *t*-tests, with the mean number of recovered larvae from the groups as the dependent variable.

### 2.8. Obtaining Filtrates from Liquid Cultures of Nematophagous Fungi

**Figure 2:**
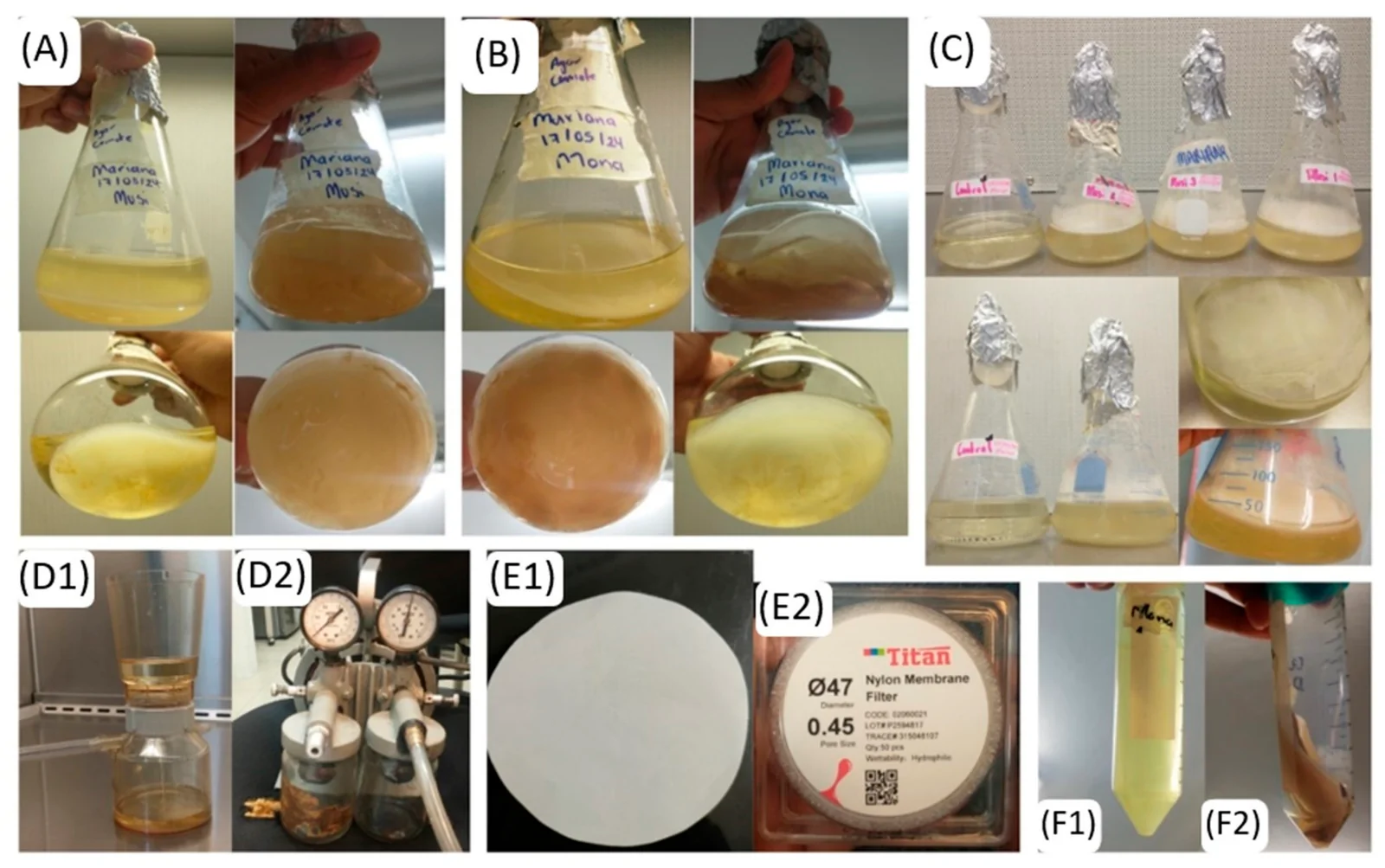
Views of two nematophagous fungi (BCh and LF isolates) cultivated in sweet potato broth ((**A**) and (**B**), respectively) and in Czapek–Dox broth (**C**) after 21 days of incubation. (**D1**) Filtration equipment, (**D2**) vacuum pump, (**E1**) Whatman filter N° 42, (**E2**) nylon membrane filter No 0.45, (**F1**) medium after filtration, (**F2**) recovered fungal biomass.

Nematophagous fungi were initially cultured on 2% bacteriological agar. After 7 days, three agar plugs (1 cm diameter) containing actively growing fungus were transferred into 250 mL flasks (*n* = 3) containing 50 mL of CzDx or SPD liquid medium (CD: 200 g sweet potato + 10 g/L dextrose). An additional flask containing only medium served as a control. All cultures were incubated for 21 days at 18–25 °C. Following incubation, the cultures were filtered to separate the mycelium from the liquid phase using a sequential filtration process: (1) coffee filter, (2) Whatman No. 42 filter (Citiva, Marlborough, MS, USA) and (3) 0.45 μm filter (Adamas-Beta, Shanghai, China). The filtrate was collected and concentrated using a rotary evaporator (40 mbar, 90 rpm; Büchi R-300, Flawil, Switzerland). The resulting concentrate was then freeze-dried with a Labconco freeze dryer (Kansas, MO, USA) (Figure 2).

### 2.9. Evaluation of the Nematicidal Activity of Liquid Culture Filtrates of Nematophagous Fungi

**Figure 3:**
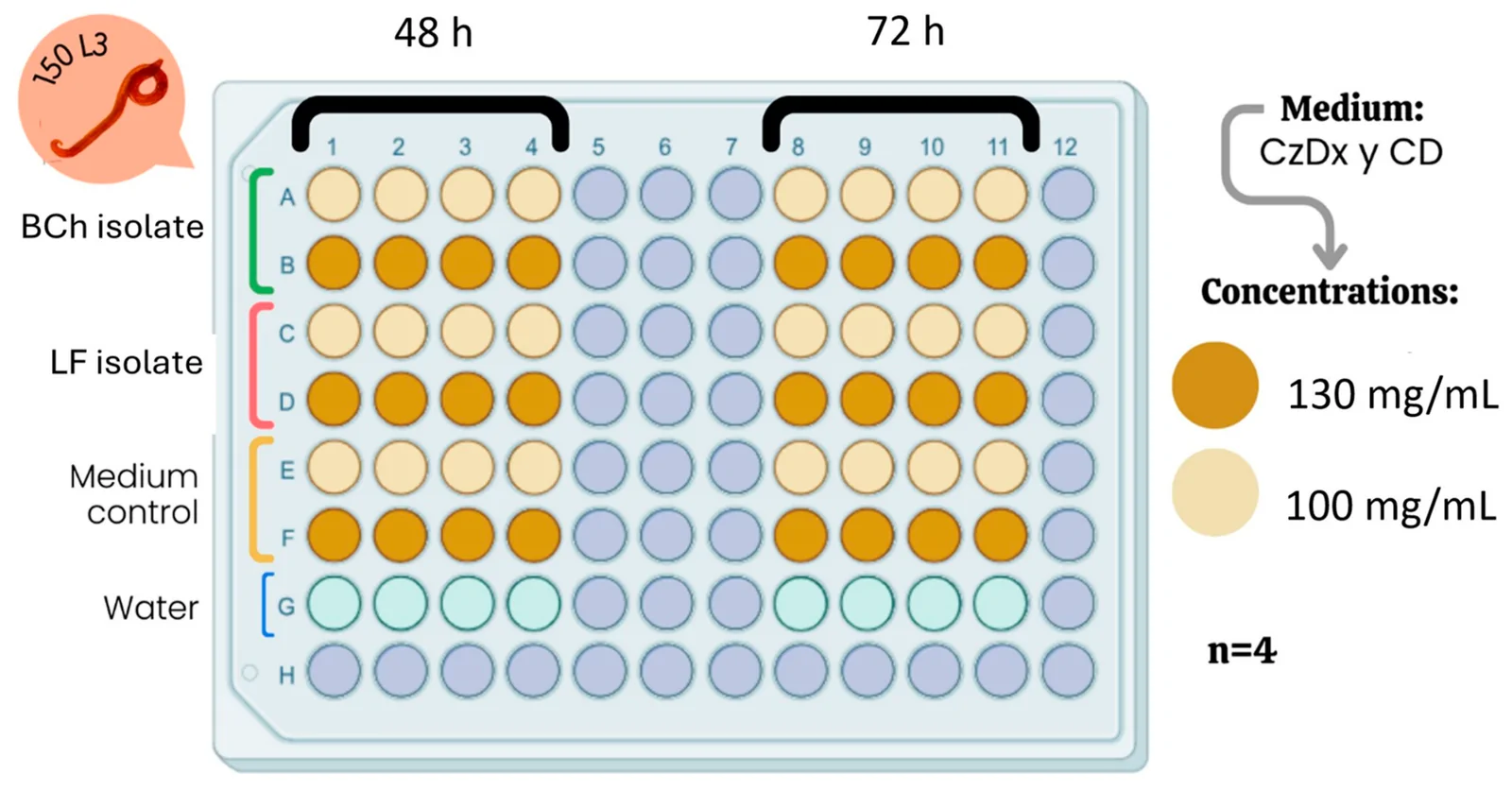
Schematic representation of the 96-well microtiter plate used to evaluate the nematicidal activity of fungal liquid culture filtrates against *Haemonchus contortus* infective larvae at two times of lectures: 48 and 72 h at two different concentrations in two liquid media.

The nematicidal activity of LCFs was assessed using a microtiter plate assay. LCFs derived from CzDx and SPD broth cultures were evaluated against *H. contortus* infective larvae, as illustrated in Figure 3. In each well, 20 microliters of an aqueous suspension containing 150 *H. contortus* infective larvae were deposited along with 80 microliters of the LCFs containing the corresponding concentration (100 and 130 mg/mL). Appropriate controls—water, CzDx broth, and sweet potato broth—were included for each treatment. The evaluations were performed at 48 and 72 h post-exposure (Figure 3). To quantify live and dead larvae, 10 drops of 10 μL each from the experimental wells were placed on a microscope slide and examined under a compound microscope at 5× and 10× magnification. Counts of live and dead larvae were recorded [31], and the mortality percentage was calculated accordingly. Each treatment consisted of four replicates, and the entire experiment was performed in triplicate for statistical analysis. A completely randomized analysis of the data was conducted using an ANOVA. The number of live and dead larvae across all treatments was the dependent variable. Mean comparisons were performed using the Tukey post hoc test. Statistical analyses were carried out with SAS 9.0 software.

## 3. Results

### 3.1. Fungal Isolation

**Figure 4:**
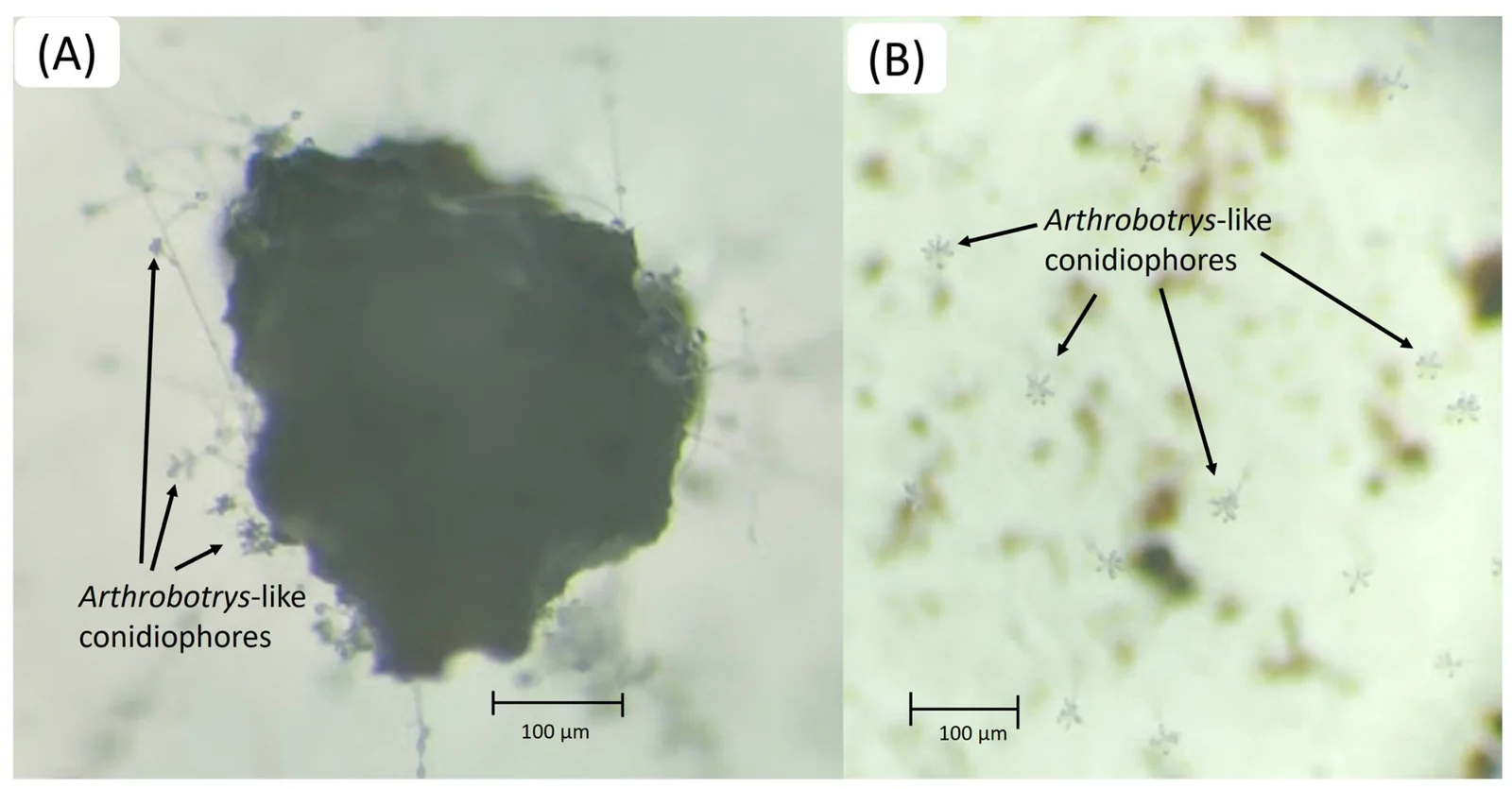
(**A**) View of a soil fragment on the surface of a water agar plate from a stereomicroscope showing the development of conidiophores typical of nematophagous fungi belonging to the genus *Arthrobotrys* sp. (LF isolate); (**B**) *Arthrobotrys*-like conidiophores emerging from the agar sprinkled with soil (BCh isolate).

Aerial structures typical of the genus *Arthrobotrys* (conidiophores) were seen under the stereomicroscope (Leitz, Wetzlar, Germany). One of the plates corresponded to the sampling performed from the Barranca de Chapultepec (BCh isolate) and the other from the Fuentes (LF isolate) (Figure 4). These structures were transferred to new sterile water agar plates to continue with the isolation process.

### 3.2. Macroscopic Comparison of Both Fungal Isolates

**Figure 5:**
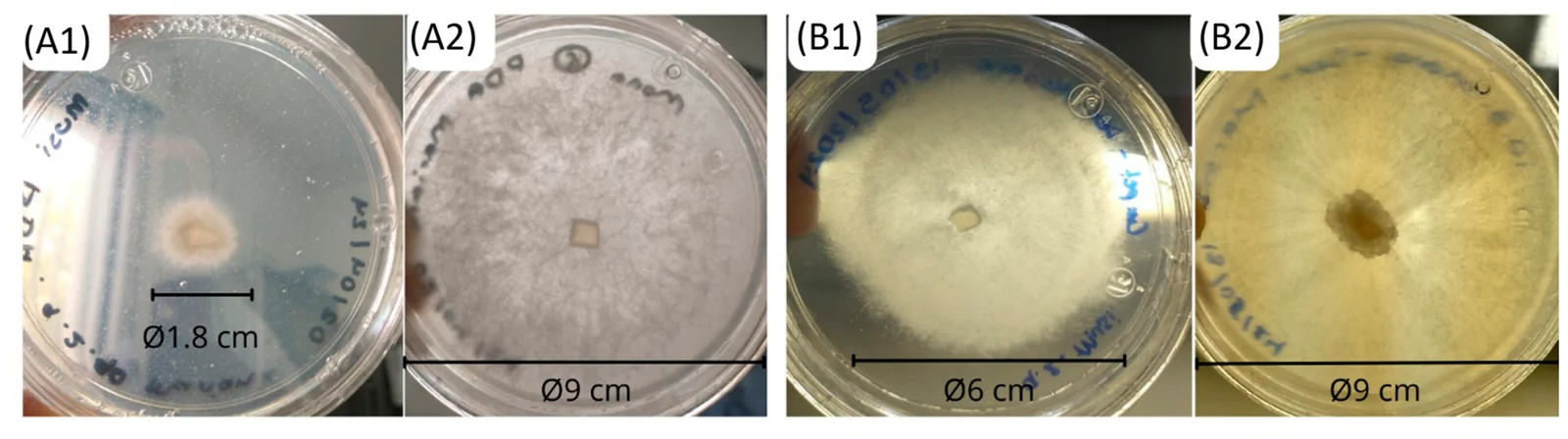
Radial growth of two nematophagous fungi isolates incubated in two culture media. (**A1**) and (**B1**): BCh isolate in Czapek–Dox and PDA, respectively. (**A2**) and (**B2**): LF isolate in Czapek–Dox and PDA, respectively.

The LF and BCh fungal strains cultivated on PDA and Czapek–Dox agar media exhibited distinct macroscopic characteristics and radial growth after 8 days of incubation. On PDA, the LF fungus displayed abundant, cottony, dispersed, and whitish growth (Figure 5(A1,A2)). In contrast, growth on Czapek–Dox agar was more hyaline and less abundant, and the mycelium developed superficially on the agar surface. In this isolate, LF fungus on PDA formed a concentrated mycelial ring with a circumference of 5.6 cm around the inoculum site, appearing hyaline and slightly cottony. Conversely, on Czapek–Dox agar, the growth was concentric, cottony, and hyaline, covering approximately 80% of the plate’s circumference (18.8 cm) and nearly the entire surface (Figure 5(B1,B2)).

### 3.3. Microscopic Comparison of Both Fungal Isolates

After a thorough examination of aerial structures relevant to taxonomy, we observed that isolate LF possesses single conidiophores, each bearing 1 to 4 conidia. The conidia are obovoidal, elongated, and septate, with 1 to 3 septa. The fungus developed three-dimensional adhesive nets in the presence of nematodes. We also visualized the presence of chlamydospores. The BCh isolate developed single, erect conidiophores with long stems bearing clusters of 8 to 10 conidia. Conidia are elongate-obovoidal to ellipsoidal, and some appeared slightly curved or straight. Chlamydospores were present. In the presence of nematodes, this isolate developed three-dimensional adhesive nets. Table 1 summarizes the characteristics of the isolates, presenting the average and range measurements of key aerial structures of taxonomic importance.

**Figure 6:**
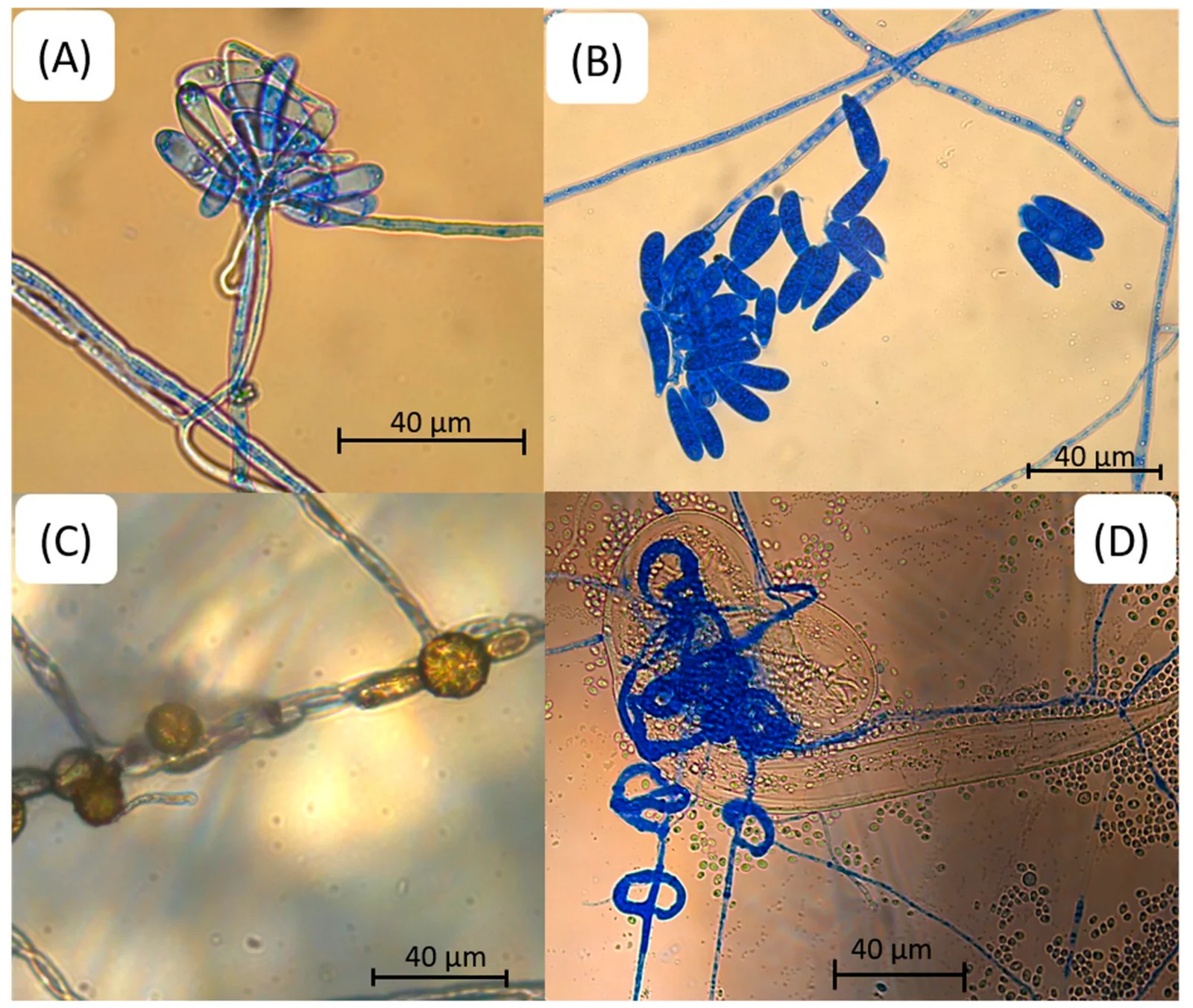
Photomicrographs illustrating key taxonomic structures of the BCh isolate *Arthrobotrys* sp. (**A**) Apical portion of a conidiophore with a cluster of conidia; (**B**) conidia; (**C**) chlamydospores; and (**D**) infective larvae of the nematode *Haemonchus contortus* trapped in a three-dimensional ring net.

**Figure 7:**
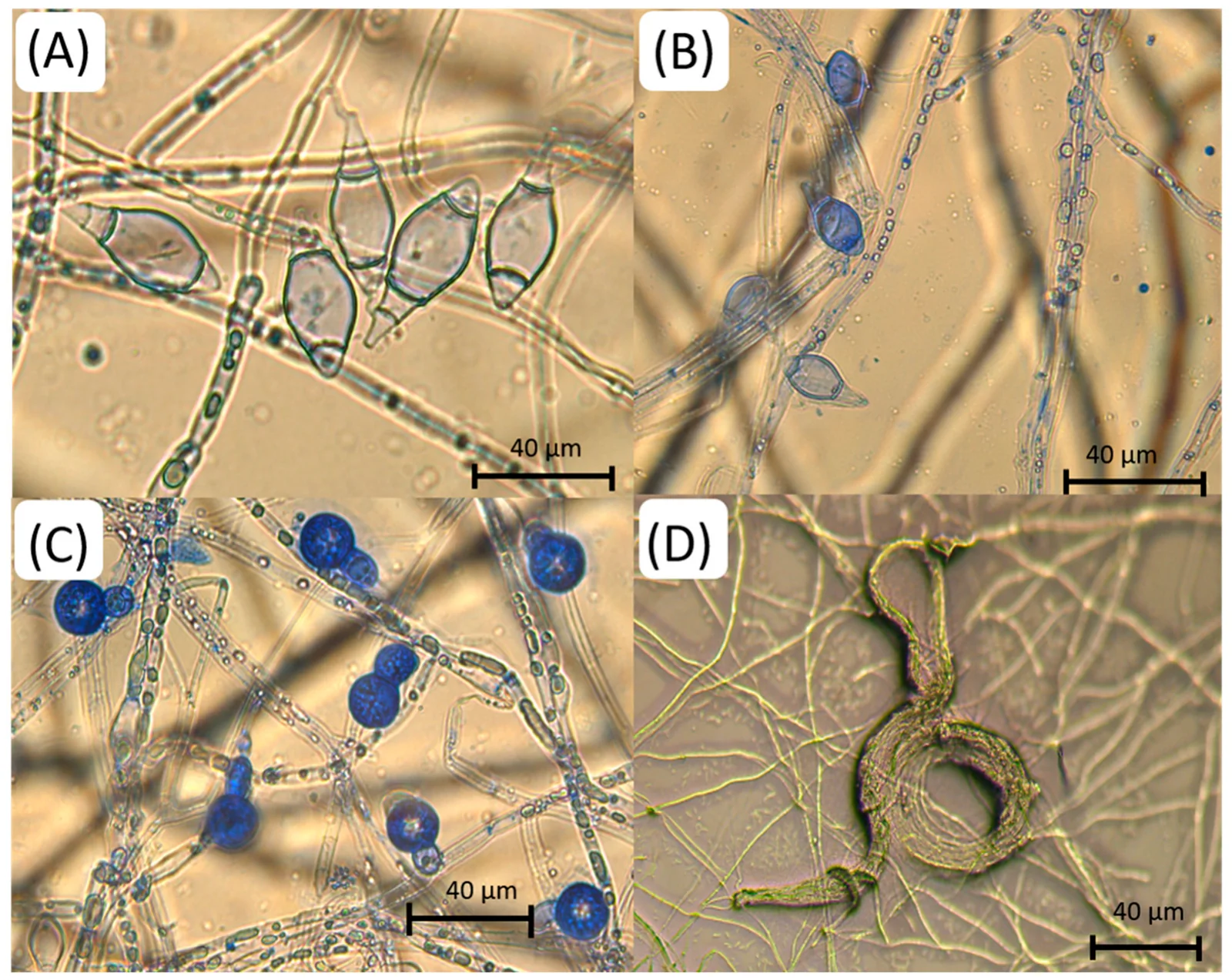
Photomicrographs illustrating key taxonomic structures of the LF isolate *Arthrobotrys* sp. (**A**) Apical portion of a conidiophore with a cluster of conidia; (**B**) conidia; (**C**) chlamydospores; (**D**) infective larvae of the nematode *Haemonchus contortus* ensnared in a three-dimensional ring net.

Figure 6 and Figure 7 present a series of microphotographs highlighting the key morphological structures of the BCh and LF isolates, respectively. The main serial structures of taxonomic importance shown in these images are conidia, conidiophores, and nematodes trapped in the fungus-trapping devices.

Based on the morphological and morphometric characteristics observed in the *Arthrobotrys* sp. and the consulted taxonomic identification guides, the evidence suggests that the bch isolate obtained from Barranca de Chapultepec is *A. musiformis*. In addition, the morphometric analysis of the second isolate (LF isolate) confirms its identification as *A. thaumasia*.

### 3.4. Molecular Identification of Arthrobotrys spp.

Sequence alignment in BLAST (NCBI) using the ITS region DNA of the isolate (Table 2) revealed 99.83% similarity with *Orbilia* (synonym *Arthrobotrys*) *multiformis* and 99.82% similarity with *Arthrobotrys musiformis*.

**Figure 8:**
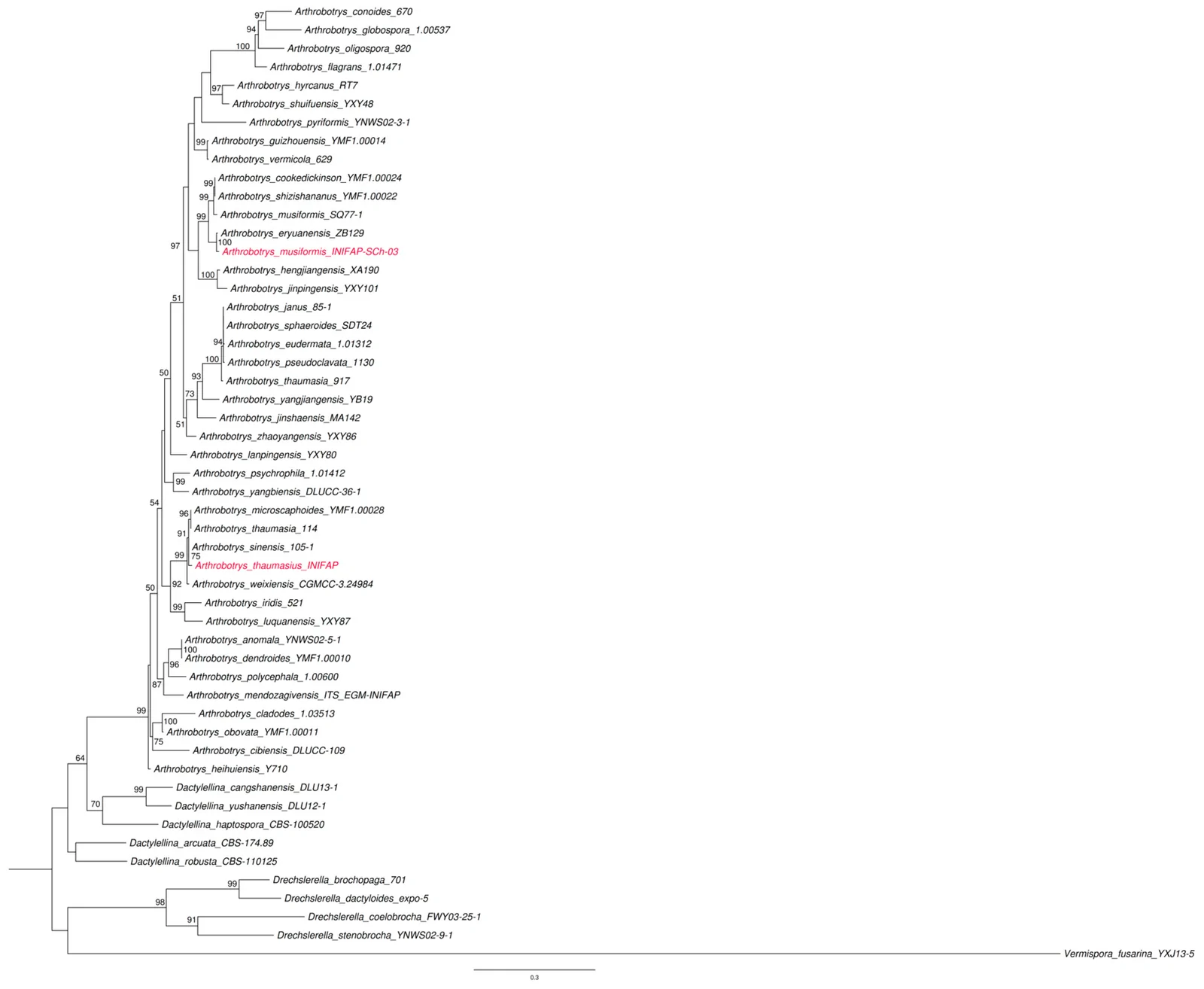
Phylogenetic tree constructed by Maximum Likelihood method, using ITS 1, 5.8S and ITS 2 sequences from strains reported in the NCBI database. Note: The *Arthrobotrys musiformis* INIFAP-Ch-03 (PQ775558) and the *A. thaumasia* Z1 LF (PQ774859) sequence are highlighted in red. Node values represent support values from the Ultrafast Bootstrapping algorithm; only values greater than 0.5 are shown.

Phylogenetic analysis was conducted using fungal DNA sequencing, which revealed a high degree of similarity to sequences in the NCBI database. To discriminate between the two isolates, which were quite similar from a molecular perspective, we additionally employed morphological taxonomic diagnostic analysis. The combination of the two taxonomic strategies allowed us to identify the BCh isolate obtained from Barranca de Chapultepec Park as *A. musiformis*. The DNA sequence was registered in the NCBI database under the name *Arthrobotrys_musiformis*_INIFAP-SCh-03 (GenBank accession number PQ775558). Based on these findings, a phylogenetic tree (Figure 8) was constructed using the Maximum Likelihood algorithm and Bayesian inference.

### 3.5. Predatory Activity of the Arthrobotrys musiformis and A. thaumasia Strains Against the Larvae (L3) of Haemonchus contortus

The number of *H. contortus* parasite larvae recovered from plates where interaction with nematophagous fungi occurred, as well as from control plates, was recorded. Table 3 presents the average number of larvae recovered from each group in which the fungi and larvae interacted, as well as from the control group. The evaluated strain of *A. musiformis* demonstrated a predation rate of 74.64%, while the *A. thaumasia* strain achieved a predation rate of 83.53%.

### 3.6. Nematicidal Effects of Liquid Culture Filtrates from Arthrobotrys musiformis and A. thaumasia Strains on Third-Stage Larvae (L3) of Haemonchus contortus

Table 4 presents the mortality percentages of *H. contortus* attributed to the lethal effect of the fungal filtrates grown in Czapek–Dox broth and in sweet potato dextrose broth. Notably, the *A. musiformis* strain exhibited strong nematicidal activity in Czapek–Dox medium, with mortality rates of 51% and 76.7% at concentrations of 100 mg/mL and 130 mg/mL, respectively, after 72 h. In contrast, in the sweet potato dextrose broth medium, the same strain showed lower mortality, with the highest mortality rates of 25.8% and 28.8% at 100 and 130 mg/mL, respectively, after 72 h. The mortality percentages occasioned by the liquid culture filtrates of *A. thaumasia* in the two liquid media were very low (<23%).

## 4. Discussion

### 4.1. Isolation and Molecular Characterization of the Isolate BCh of Arthrobotrys musiformis

Analysis of the genetic sequences from this isolate, along with the alignment data, revealed high coverage and similarity percentages compared to 27 sequences previously reported in GenBank (NCBI). These results indicate a strong phylogenetic relationship with the species *Arthrobotrys* (*Orbilia*) *multiformis*, *A. eryuanensis*, and *A. musiformis*; however, the phylogenetic tree generated shows a closer relationship with *A. musiformis*. This finding complements traditional taxonomy by highlighting the morphological and morphometric characteristics that distinguish this fungus from other phylogenetically similar isolates. The phylogenetic tree was constructed using 52 strains: 42 of *Arthrobotrys* (including our own), 5 of *Dactylellina*, and 4 of *Drechslerella*, with *Vermispora fusarina* as the outgroup. Morphological analyses revealed strong similarities between our strain and both *A. musiformis* and *A. thaumasia*. Notably, *A. musiformis* develops erect, candelabra-like conidiophores (104–640 µm, occasionally up to 900 µm), each bearing apical conidia in clusters of 5–15. The conidia are hyaline, ellipsoidal, and straight or slightly curved, with a basal septum, measuring 20–45 µm in length and 7–12.5 µm in width. Additionally, yellowish, globose chlamydospores (14–22 µm) are observed in older cultures. These features closely correspond to our strain’s observations and measurements. NCBI-BLAST alignment further demonstrated high sequence similarity to *A. multiformis* (99.83%) and *A. musiformis* (99.12%). However, key morphological differences distinguish these species: *A. musiformis* exhibits conidiophores up to 640 µm tall with clusters of more than five one-septate conidia, while *A. multiformis* has conidiophores up to 220 µm tall, each topped by a single multiseptate conidium. The conidia of *A. musiformis* are ellipsoidal and slightly curved (20–47.5 × 7–12.5 µm), whereas those of *A. multiformis* are elongated-fusiform with 4–12 septa (47–198 × 7–20 µm; [32,33]. Similarly, phylogenetic analysis placed our *A. musiformis* strain in a clade with *A. cookedickinson*, *A. shizishananus*, *A. musiformis*, and *A. eryuanensis*. Nonetheless, *A. cookedickinson* and *A. shizishananus* possess conidiophores with a single apical conidium that is 2–3 septate and 3–7 septate, respectively [33], while *A. eryuanensis* is differentiated by branched conidiophores and the production of both macro- and microconidia [26]. In terms of morphological characteristics, *A. thaumasia* displays erect conidiophores measuring 195–460 µm in length, which are typically simple but may occasionally branch, each bearing clusters of 3 to 15 conidia. The conidia themselves are hyaline and top-shaped, featuring rounded apices and 1–4 septa. The fungus also forms yellowish, globose-to-ellipsoidal chlamydospores and captures nematodes using adhesive, three-dimensional networks. The features observed and measured in this study are consistent with previous reports [32,33]. Additionally, NCBI-BLAST alignment showed high similarity to strains of *A. thaumasia* (98.03–99.83%) and *A. sinensis* (98.32%). However, *A. sinensis* produces sub-spherical or obovoid conidia that are smaller (<30 µm long). Phylogenetic analysis further placed our strain in the same clade as *A. microscaphoides*, which is distinguished by cymbiform conidia with 0–3 septa, most commonly two. Presented below are certain strains of *A. musiformis* and *A. thaumasia* from various geographic regions that exhibit the highest coverage and similarity values relative to the isolates from our study; a high degree of similarity is evident among the strains reported in GenBank, thereby supporting the taxonomic identification. It is worth noting that there are minimal differences (<3%) among the reported strains when the ITS region is used. While analyzing a larger DNA region would allow for better differentiation between strains, the morphological and molecular analyses robustly support the identification. Similarly, an analysis of ITS sequences across different *A. thaumasia* strains reveal very low divergence (<3%) from the strain reported in this study, despite their isolation from different countries; consequently, a more in-depth phylogenetic analysis would benefit from the inclusion of additional regions. Nevertheless, complementing the molecular analysis with the fungus’s morphology enables its differentiation from other similar species.

Comparative tables showing *A. musiformis* and *A. thaumasia* strains from different countries previously reported at the GenBank, their bank accession numbers, their query cover and their similarity are shown below (Table 5 and Table 6).

### 4.2. Assessment of the Predatory Activity of Arthrobotrys musiformis and A. thaumasia

The fungi studied demonstrated notable predatory activity against the nematode *H. contortus* (L3), with both strains exhibiting high predation rates. The *A. musiformis* strain, isolated from Chapultepec Park in Cuernavaca, Morelos, achieved a predation rate of approximately 75% after 10 days of exposure. In comparison, the *A. thaumasia* strain displayed an even higher rate, reaching 84% predation against *H. contortus* larvae (L3). These findings are significant and align with previous research involving other isolated strains. For instance, a recent study utilizing *A. musiformis* obtained from soil in Cuautla, Morelos, Mexico, reported a 70% predation rate against infective *H. contortus* larvae [29]. Other isolates of *A. musiformis* have demonstrated a wide range of predatory activity. For instance, several isolates collected from different regions in Tabasco exhibited 100% predatory activity against fungi sourced from the Bajío and Yumka localities, while an isolate from Macuspana, Tabasco, Mexico, showed 55.5% predatory activity after 7 days of incubation [34]. These results suggest that both the site of fungal isolation and prevailing environmental conditions at the time of collection may play a significant role. Additionally, a 74.9% predatory capacity was reported for an *A. musiformis* isolate obtained from the Chapultepec Ecological Park in Cuernavaca, Morelos [35]; likewise, another author reported a predation rate of 70.95% against the same nematode using a fungus of this species isolated from soil at a poultry farm in Cuernavaca, Morelos. Generally, predation rates across isolates of this species are approximately 70%, indicating their potential as biological control agents against important livestock parasites [36]. In the present study, the fungus *A. thaumasia* exhibited 85% predatory activity. This species has been rarely reported, and only a few studies on it are available. For instance, it has been observed that the predatory activity of this species ranged from 75.54% to 99.97% against trichostrongylid larvae, including *Haemonchus contortus*, *Trichostrongylus colubriformis*, and *Marshallagia mongolica* [37]. A variety of biotic and abiotic factors can influence the spore germination and predatory activity of nematophagous fungi. These include the microenvironment, shaped by soil microfauna, micronutrients, pH, humidity, and temperature [38]. Such factors may increase nematode populations and promote a shift in fungi from saprophytic to predatory behaviour by providing a carbon and nitrogen source suitable for nutrient uptake from nematodes. This, in turn, can indirectly boost the population of nematophagous fungi [39,40]. Table 7 presents a comparative overview of nematophagous fungi species, the target nematodes, experimental conditions, and the results of their predatory activity.

### 4.3. Nematicidal Activity of Liquid Filtrates from the Arthrobotrys musiformis and A. thaumasia Strains

The liquid filtrates evaluated in this study demonstrated clear nematicidal activity, with both *A. musiformis* and *A. thaumasia* strains exhibiting lethal effects against infective *Haemonchus contortus larvae*. Notably, the *A. musiformis* strain achieved the highest lethality at 72 h, with mortality rates of 51% and 76% in Czapek–Dox medium at concentrations of 100 and 130 mg/mL, respectively. These findings suggest that the filtrates possess bioactive compounds responsible for nematicidal action. In comparison, the liquid filtrates derived from sweet potato dextrose cultures of the *A. thaumasia* strain induced mortality rates ranging from 25% to 30% when tested at 130 mg/mL at 48 and 72 h. Previous studies have shown that nematophagous fungi synthesize a diverse array of nematicidal compounds [45], with species such as *Orbilia oligospora* being particularly prolific producers [46]. Regarding the metabolite production, both quantity and quality depend on a number of factors, such as the amount and quality of nutrients in the medium, the presence of oxygen and the presence of nematode components that act as nematocide compound elicitors [47]. Previous research demonstrates that the species *A. musiformis* and *A. thaumasia* possess notable nematicidal activity and a strong ability to synthesize compounds that immobilize and kill the nematode *H. contortus*.

Secondary metabolites produced by *A. musiformis* exhibit significant lethal activity, with the specific compounds produced varying based on the nutrients available in the growth medium. For example, cultivating this species in Czapek–Dox broth yielded alkaloids, saponins, and coumarins that were highly effective against *H. contortus* (>90%) [29]. Proteolytic enzymes were detected in potato dextrose broth after 14 days at 25 °C by ion-exchange chromatography. These enzymes damaged the *H. contortus* sheath, 634 resulting in 77% mortality [48]. In a related study, liquid filtrates from *A. musiformis* cultured in potato dextrose broth for 7 days at 26 °C reduced the number of infective larvae of *H. contortus*, *Trichostrongylus colubriformis*, and *Marshallagia mongolica* by 75.54% to 99.97% [37]. Several compounds with nematicidal activity have been identified in *A. thaumasia*, including trimethyl-heptadiene, methyl-hexadecanol, dodecadienal, decane, terpendol E, dodecane, acetamido-6-anthraquinone, and hexadecanol. These compounds demonstrated substantial activity—exceeding 56%—against the plant-parasitic nematode *Meloidogyne incognita*. Identification was achieved by incubating *A. thaumasia* in potato dextrose broth for 10 days at 25 °C with constant agitation at 180 rpm [49]. Furthermore, the presence of chitinases was confirmed by incubating an isolate of this species on cornmeal agar for 5 days at 28 °C. This enzyme exhibited significant activity against the free-living nematode *Panagrellus redivivus*, resulting in 80% mortality [50]. Two comparative tables summarize the production of enzymes and metabolites identified in various nematophagous fungi exhibiting antagonistic activity against different nematode species, as shown in Table 8 and Table 9, respectively.

It is important to consider that nematophagous fungi offer two practical alternative methods for controlling gastrointestinal parasitic nematodes. One approach involves mixing fungal spores with animal feed so that some spores reach the fecal matter and exert their predatory activity, thereby substantially reducing larval populations and preventing grass contamination. The second method explores the use of natural nematocidal compounds found in fungal liquid culture filtrates, which act as natural anthelmintics to reduce fecal egg counts and further grass contamination.

## 5. Conclusions

This study isolated and identified, both morphologically and molecularly, an isolate of the nematophagous fungus *Arthrobotrys musiformis* as well as another belonging to *A. thaumasia*. The predatory activity of these isolates and the nematicidal activity of their liquid filtrates against *Haemonchus contortus* were evaluated. Global research on *A. thaumasia* remains limited, yet recent studies highlight its promising predatory activity and the potency of its liquid filtrates. Here, we report novel findings on an isolate of this species that, although it did not exhibit high lethal activity in its culture filtrates, demonstrated its potential as a biological control agent against ovine haemonchosis in forthcoming trials due to its high predatory activity. The liquid culture filtrates derived from *A. musiformis* exhibited strong nematicidal activity against the infective larvae of *H. contortus*. This finding highlights its potential as a promising candidate for further research to identify the specific compound(s) responsible for this effect and to discover novel nematicidal natural compounds.

## Figures and Tables

**Table 1:** Average and range measurements for conidia and conidiophores, measurements from the base to the septum and the presence of chlamydospores of the fungi *Arthrobotrys* sp. (“BCh” isolate) and *Arthrobotrys* sp. (“LF” isolate).

	*Arthrobotrys* sp. (BCh Isolate)		*Arthrobotrys* sp. (LF Isolate)	
Characteristics	Average (µm)	Range (µm)	Average (µm)	Range (µm)
Conidium length	31.60	24.54–38.55	36.14	28.49–43.8
Conidium width	12.58	8.13–17.87	17.24	13.92–21.56
Measurement from the base to the septum	9.44	6.32–12.71	12.5	8.91–16.1
Conidiophore length	48.70	10.05–86.75	365	330–400
Chlamydospores	+		+	

The symbol “+” indicates presence of chlamydospores.

**Table 2:** Similarity and coverage results for the isolated ITS sequence after comparison with sequences registered in the GenBank-NCBI database.

Strain	Covering %	Similarity %	GenBank Accession Number
*Arthrobotrys musiformis* BCh ^(a)^			PQ775558
*Orbilia multiformis* FA626	100	99.83	OM066034.1
*Orbilia multiformis* FA829	100	99.83	OM066033.1
*Orbilia multiformis* FA639	100	99.83	OM066016.1
*Orbilia multiformis* FA804	100	99.83	OM066042.1
*Orbilia multiformis* FA736	100	99.83	OM066041.1
*Arthrobotrys musiformis* FA761	99	99.83	OM660023.1
*Arthrobotrys musiformis* Mau1Ad	96	99.12	MN831892.1
*Arthrobotrys thaumasia* Z1-LF ^(b)^			PQ774859
*Arthrobotrys thaumasia* isolate 110	97	99.3	EU977529.1
*Arthrobotrys thaumasia* isolate 111	96	99.83	EU977532.1
*Arthrobotrys thaumasia strain* CBS 322.94	95	99.32	AF106526.1
*Arthrobotrys thaumasia strain* CBS37697	99	98.03	KT215216.1
*Arthrobotrys thaumasia* Isolate BUAT-AT-2	99	97.87	OR859125.1
*Arthrobotrys thaumasia isolate* I-Y4-2	93	99.82	PP319009.1
*Arthrobotrys sinensis strain* 105-1	97	98.32	AY773445.1

Note: (a) and (b) correspond to the isolates used in the present study.

**Table 3:** Predation percentages against the nematode *Haemonchus contortus* after 10 days of confrontation of the *Arthrobotrys musiformis* and *A. thaumasia* strains.

Treatment	Mean of Recovered Larvae	Predation (%)
*Arthrobotrys musiformis*	710 ± 342.7	74.64 ± 4.2 *
Control without fungi	2800 ± 830.2	
*A. thaumasia*	227.7 ± 86.2	83.53 ± 4.5 *
Control without fungi	1383 ± 142.9	

Paired *t*-test. * = statistically significant difference. Note: (±) standard deviation, n = 3; *p* ≤ 0.023.

**Table 4:** In vitro mortality percentages of *Haemonchus contortus* infective larvae (L3) following treatment with liquid culture filtrates derived from two nematophagous fungal strains, *Arthrobotrys musiformis* and *A. thaumasia*, produced in two media at two different times.

	Fungi Growing in Czapek–Dox Broth		Fungi Growing in Sweet Potato Dextrose Broth	
Liquid Culture Filtrate/Concentration	Evaluation Time (h)		Evaluation Time (h)	
	48	72	48	72
*A. musiformis* 100 mg/mL	30.3 ± 3.7 ^a^	51.0 ± 14.7 ^b^	7.8 ± 6.9 ^ab^	25.8 ± 19.1 ª
*A. thaumasia* 100 mg/mL	15.5 ± 8.5 ^b^	12.3 ± 1.6 ^c^	5.8 ± 2.9 ^ab^	17.6 ± 4.4 ^ab^
*A. musiformis* 130 mg/mL	32.0 ± 7.8 ^a^	76.7 ± 13.5 ^a^	10.3 ± 6.3 ^bc^	28.8 ±12.1 ^a^
*A. thaumasia* 130 mg/mL	15.0 ± 1.2 ^b^	22.4 ± 2.1 ^c^	8.3 ± 2.4 ^bc^	21.0 ± 4.3 ^ab^
Czapek–Dox	4.8 ± 0.9 ^c^	6.5 ± 4.3 ^c^	3.5 ± 1.2 ^bc^	5.4 ± 3.8 ^bc^
Water	1.1 ± 0.4 ^c^	1.9 ± 1.5 ^c^	0.9 ± 0.7 ^c^	1.0 ± 0.4 ^c^

Tukey test: Means with different letters are statistically different. Values shown are mean ± standard deviation. Statistical significance was set at *p* ≤ 0.05 (n = 3).

**Table 5:** GenBank accession, query cover and similarity of *Arthrobotrys musiformis* strains from different countries.

Strain Code	Country	GenBank Accession Number	Query Cover (%)	Identity (%)
AmusichapudeGives	Mexico	OR195437.1	100	97.27
CBS 110.37	USA	MH855842.1	100	97.27
3Y7A-1-1	China	OL454931.1	100	97.27
INIFAP-SCt-01	Mexico	PP333206.1	100	97.27
	USA	KJ938572.1	100	97.10
Am_11	Portugal	MG515529.1	100	97.1
SQ77-1	China	AY773469.1	100	94.06
FB01	Mexico	MF926584.1	99	97.26
106	China	EU977517.1	99	97.09
INIFAP-SCv-02	Mexico	PP567276.1	98	97.40

**Table 6:** GenBank accession, query cover and similarity of *Arthrobotrys thaumasia* strains from different countries.

Strain	Country	GenBank Accession Number	Query Cover (%)	Identity (%)
110	China	EU977529.1	97	99.33
111	China	EU977532.1	96	99.83
CBS 322.94	Germany	AF106526.1	95	99.32
CBS 376.97	Burkina Faso	KT215216.1	99	98.03
BUAT-AT-2	India	OR859125.1	99	97.87
I-Y4-2	Turkey	PP319009.1	93	99.82
BHAT-AT-1	India	OR859119.1	99	97.55
At_RK	India	MT898981.1	96	98.46
NBS005	China	KX640093.1	94	98.95
1.03171	China	MH179771.1	96	98.12

**Table 7:** Comparative table showing the predatory activity of various *Arthrobotrys* species evaluated under different experimental conditions and against different blank nematodes, with results presented as percentages of predatory activity.

Arthrobotrys Species	Blank Nematode	Conditions	Predatory Activity	Authors
*Arthrobotrys cladodes var. macroides*	*Haemonchus contortus*	In vitro assessment of reduction in infective larvae in sheep feces	At 20 × 10^3^ conidia = 73.49%; at 100 × 10^3^ conidia = 94.96%	[41]
*A. robusta*	*Haemonchus placei*	In vivo assessment administering 2 × 10^6^ conidia (per os) in calves	73.68%	[42]
*A. thaumasia*	*Trichostrongylus colubriformis*, *Haemonchus contortus*, and *Marshallagia mongolica.*	In vitro assessment of fungus spores (after passing through sheep gastrointestinal tract)	75.54–99.97%	[37]
*Duddingtonia flagrans*	*Trichostrongylus colubriformis*	In vitro with 16 native isolates from China	57.21–99.83	[43]
*A. oviformis* *A. sinense* *A. microscaphoides*	Mixture of genera/species in sheep *Haemonchus contortus* *Trichostrongylus colubriformis*	Oral doses	*A. oviformis* = 84.12% *A. sinense* = 84.39% *A. microscaphoides* = 84.74%	[44]

**Table 8:** A comparative analysis of enzymes identified in various nematophagous fungi exhibiting diverse nematode-antagonistic activities.

Protein Group	Fungal Species	Nematode Blank	Activity	Reference
Serine-dependent proteases	*Arthrobotrys musiformis*	*Haemonchus contortus*	A time-dependent effect was identified, with 77% larvae immobilization after 48 h of incubation.	[48]
Serine proteases AmSP1	*A. musiformis*	Various nematodes	The gene responsible for the cuticle-degrading serine protease was identified.	[51]
Fitase BPP	*Arthrobotrys oligospora*	Various nematodes	A high specific enzymatic activity (74.71 U/mg) was identified during the development of three-dimensional adhesive nets.	[52]
Serine proteinase P186	*oligospora*	Various nematodes	The enzyme was found degrading different substrates: casein, gelatin, bovine serum albumin, denatured collagen, and the cortical layer of nematodes.	[53]
Chitinase AO-801	*A. oligospora*	*Caenorhabditis elegans*	The enzyme displayed activity degrading colloidal and powdered chitin, egg lysate, and L1 larva lysate of *Caenorhabditis elegans*.	[54]
Recombinant chitinase AO-379	*oligospora*	*Strongylus equinus*, *Caenorhabditis elegans* and *Haemonchus contortus*	The degradation activities on the nematodes were taken at 12, 24, and 36 h; the activities were as follows: *S. equinus*: 42%, 89%, and 100%; *C. elegans*: 50%, 90%, and 97%; *H. contortus*: 53%, 62%, and 84%.	[55]
Protease chitinase	*D. flagrans*	*Cyathostomins*	The enzyme reduced the L3 Cyathostomins percentages as follows: protease = 19.4%, chitinase = 15.5%, protease + chitinase = 20.5%.	[56]

**Table 9:** A comparative analysis of compounds derived from the secondary metabolism of different nematophagous fungi exhibiting diverse nematode-antagonistic activities.

Metabolite	Fungal Species	Blank Nematode	Antagonistic Effect	References
4-(4′-carboxy-2′- ethylhydroxypentyl)- 5,6-dihydro-6- methylcyclobuta[b] pyridine3, 6-dicarboxylic acid	*Paecilomyces* sp.	*Panagrellus redivivus*, *Meloidogyne incognita* and *Bursaphelenchus xylophilus*	*P. redivivus:* LD50: 50.85 mg/mL *M. incognita*: LD50: 47.1 μg/mL *Bursaphelenchus xylophilus*: LD50 at 167.7 μg/mL	[57]
Aurovertins A3 y A4	*Pochonia clamydosphoria*	*M. Incognita* (J2)	Nematode suppression at 48 h: Aurovertins A3: LD50: 88.6 μg/mL Aurovertins A4: LD50: 41.7 μg/mL)	[58]
Roselipinas 1–5	*Clonostachys candelabrum*	*Haemonchus contortus* (L3)	Motility inhibition = 90% At 22 µg/mL	[59]
2 (5H)-Furanone, 5-methylfuran-2-carbaldehyde (compound 8)	*Arthrobotrys oligospora*	*Caenorhabditis elegans*	Nematode attraction index: 0.4 Paralyse and kill nematodes at LC50: 369 μg/mL in 12 h.	[38]

## Data Availability

The original contributions presented in this study are included in the article. Further inquiries can be directed to the corresponding author(s).
